# Management of Pilon Fractures—Current Concepts

**DOI:** 10.3389/fsurg.2021.764232

**Published:** 2021-12-23

**Authors:** Olivia Mair, Patrick Pflüger, Kai Hoffeld, Karl F. Braun, Chlodwig Kirchhoff, Peter Biberthaler, Moritz Crönlein

**Affiliations:** ^1^Department of Trauma Surgery, Klinikum rechts der Isar, Technical University of Munich, Munich, Germany; ^2^Center for Orthopedic and Trauma Surgery, University Medical Center, Cologne, Germany; ^3^Department of Trauma Surgery, Center for Musculoskeletal Surgery, Charité University Medicine Berlin, Berlin, Germany

**Keywords:** tibial pilon fractures, distal tibial fractures, ankle fractures, soft tissue management, tibia–injuries

## Abstract

Tibial pilon fractures were first described by Étienne Destot in 1911. He used the French word “pilon” (i.e., pestle), to describe the mechanical function of the distal tibia in the ankle joint. This term has further been used to portray the mechanism involved in tibial pilon fractures in which the distal tibia acts as a pestle with heavy axial forces over the talus basically causing the tibia to burst. Many different classification systems exist so far, with the AO Classification being the most commonly used classification in the clinical setting. Especially Type C fractures are extremely difficult to manage as the high energy involved in developing this type of injury frequently damages the soft tissue surrounding the fracture zone severely. Therefore, long -term outcome is often poor and correct initial management crucial. In the early years of this century treatment has evolved to a two–staged protocol, which nowadays is the gold standard of care. Additional methods of treating the soft tissue envelope are currently being investigated and have shown promising results for the future. The aim of this review is therefore to summarize protocols in managing these difficult fractures, review the literature on recent developments and therefore give surgeons a better understanding and ability to handle tibial pilon fractures.

## Epidemiology

Tibial pilon fractures are quite rare, accounting for ~3–10% of all tibial fractures and <1% of all fractures to the lower extremity (1–3). Men tend to suffer from these injuries slightly more often than women with the majority of injuries occurring at around 45 years (3, 4). In ~75–90% of all cases the fibula is also fractured (5). Tibial pilon fractures with the fibula intact are more likely in AO Type B fractures than in Type C fractures. Furthermore, in recent studies it has been suggested that tibial pilon fractures are likely to be less comminuted and less severe when the fibula remains intact (6).

## Etiology

In contrast to simple ankle fractures, pilon fractures usually result from high—energy trauma with heavy axial force, which basically causes the tibial plafond to burst over the talus (7). Sometimes rather low—energy rotational forces, for example in skiing accidents, can also lead to pilon fractures, but the comminution seen in these fractures is usually less severe (5). Most commonly the high–energy traumas are due to falls or jumps from great heights or motor vehicle accidents. The high energy surrounding the accidents cause severe damage to the surrounding soft tissue as well and ~6% of all patients with tibial pilon fractures have multiple injuries and require intensive care units (7, 8).

## Pathoanatomy

The position of the foot at the time of axial impact seems to be the decisive factor in terms of fracture pattern and amount of comminution (9, 10). Topliss et al. emphasize the importance of the position of the foot at the time of axial impact and therefore distinguish between fractures in the sagittal plane and those in the coronal plane. Sagittal fractures are mostly seen in younger patients and high–energy trauma with the foot in varus angulation at the time of impact, while coronal fractures are rather seen in older patients, with low–energy trauma and the foot in valgus angulation (10). Furthermore, when the foot is in plantar flexion at the time of impact the force will likely cause a fracture of the posterior part, when the foot is in dorsiflexion an anterior fracture of the tibial pilon is the result. When the foot is in neutral position at the time of the impact, the talus will act as a pestle, which will result in destruction of the whole articular surface (8).

The rate of open fractures varies greatly depending on the mechanism of the injury, with up to 50% reported in high energy traumas (11).

## Classification

Rüedi and Allgöwer were amongst the first trauma surgeons to extensively research pilon fractures. From the cohort study they conducted they derived a classification system separating pilon fractures into three different categories based on the extent of the comminution and the displacement of the articular surface. Furthermore, they formulated a treatment plan for each type of pilon fracture (12, 13).

Overall, this classification system oversimplifies description of the highly complex fracture sites in pilon fractures and therefore lacks the ability to provide sufficient support in preoperative planning. Nevertheless it lay the foundations for future classification systems (7).

In 1990 the Arbeitsgemeinschaft für Osteosynthesefragen / Orthopedic Trauma Association (AO / OTA) developed a more extensive classification system for all fractures of the body based on the Comprehensive Classification of Fractures of the Long Bones (CCF) developed by Müller et al. (14). It uses alphanumeric codes and has been reviewed and updated regularly (15). True tibial pilon fractures are classified by the code AO 43C, further numbers are added to describe the exact location, comminution and extent of the fracture (15). While the AO Classification System is generally understood worldwide, it has moderate to poor intra- and interobserver reliability (16–18). Nevertheless, this problem does not seem to be problematic in terms of outcome and quality of reduction and it has been suggested that the routinely use of 3D-imaging in these complex fractures will further enhance reliability of the classification system (7, 19).

**Figure 1:**
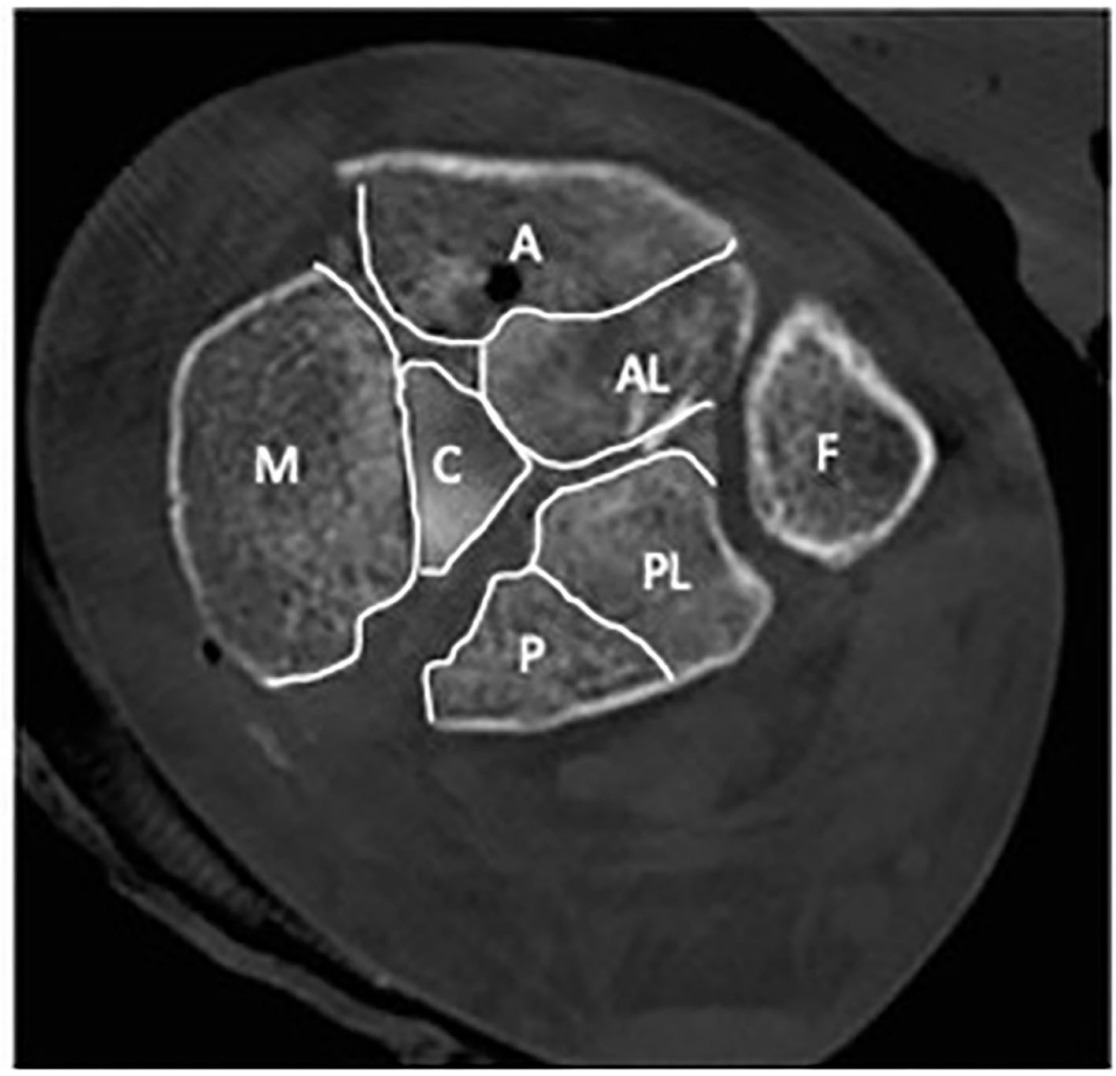
An axial CT of the distal tibia showing the typical six fracture fragments. A, anterior fragment; AL, anterolateral fragment; P, posterior fragment; PL, posterolateral fragment; C, central die-punch fragment; M, medial fragment; F, fibula.

Topliss et al. introduced a more advanced classification system using an axial CT scan to identify the six typical fragments: an anterolateral, anterior, posterior, posterolateral, medial and central die-punch fragment (Figure 1). They are present with varying frequency and need to be analyzed carefully in order to choose the appropriate approach and plate position (10).

## Management in the Emergency Setting

As 6% of all tibial pilon fractures are seen in polytraumatized patients, the patient should always be assessed after advanced trauma life support protocols (“ATLS”) in the emergency room and in the field (8). When life threatening injuries have been ruled out, obviously deformed ankle fractures should be reduced as early as possible under adequate pain management or procedural sedation. Prior to and after reduction a thorough neurovascular exam is essential. Early reduction reduces tension on neurovascular structures thus slowing the swelling of the soft tissue, usually decreases pain levels significantly and furthermore decreases the chances for further cartilage damage and tissue necrosis (20).

To achieve satisfying anatomical reduction of the fracture can be rather difficult in highly comminuted fractures or where entrapped tissue or bone renders the fracture irreducible. In these cases temporary external fixation in terms of a damage control surgery is to be done as quickly as possible to reduce the risks accompanied by extensive soft tissue damage (21, 22). After successful reduction close observation of the soft tissue is essential as vascular injuries around the ankle are commonly seen in high -energy trauma and can lead to compartment syndrome (8).

In open tibial pilon fractures the wound should always be cleaned thoroughly, cut out and close up if possible (likely grade I and II open fractures). When the loss of soft tissue is too extensive or wound contamination is too gross, radical debridement, lavage, treatment with a vacuum assisted closure (VAC) device and temporary external fixation can be necessary. In addition intravenous antibiotic therapy is essential and should be administered as early as possible (23).

## Non-operative Treatment

It is widely agreed that surgical fixation and reduction is the treatment of choice for pilon fractures (24–27). Nevertheless in an aging population sometimes surgery cannot be safely accomplished in which case the treatment consists of reduction as described above and non–weight–bearing immobilization for 6–10 weeks in a heavy cast. In light of spinal anesthesia and modern methods of treatment at least external fixation will likely be possible in most patients (8).

## Operative Treatment

Up until the end of the last century it was widely agreed that operative treatment should be achieved as early as possible to reduce the length of hospital stays and associated complications. Good outcomes were reported when this course of treatment was used for low energy injuries (Rüedi–Allgöwer I and II), but the outcomes were poor in high-energy traumas, highly comminuted and displaced tibial pilon fractures and when comorbidities in the patient were detected. Multiple individual studies showed an unacceptably high rate of infections (up to 40%) and wound complications resulting in a high rate of revision surgeries, non-union, osteomyelitis, poor functional outcome and arthritis in early open reduction and internal fixation (11, 24, 28–32). The high complication rate was mainly attributed to the severe iatrogenic trauma during open reduction to an already damaged soft tissue envelope (33).

Due to this extremely high complication rate the two-staged approach was established and is still the most commonly used treatment method for tibial pilon fractures. Numerous authors reported a significantly lower rate of complications, such as infections and non-unions and a better functional outcome in a two–staged treatment protocol (24, 28, 34, 35).

## Temporary Fixation

Tibial pilon fractures due to low energy trauma without much comminution and less severe soft tissue damage can initially be sufficiently treated with closed reduction and immobilization in a well-padded cast.

But as most tibial pilon fractures are strongly comminuted due to the high energy trauma mechanism, the initial treatment consists of ankle–spanning external fixation (=EF) in terms of a damage control surgery and delayed open reduction and internal fixation (=ORIF). Temporary fixation should be performed as quickly as possible to achieve realignment and restore length and anatomical reduction. It is important to place the pins well outside of the fracture zone in order not to interfere with definite fixation and cross future surgical approaches (36). There are multiple different external fixator systems with most of them forming an “A-figure” over the ankle. An extension of the external fixator onto the forefoot, most commonly the first metatarsal, is advisable to prevent equinus contracture (37). This secure reduction and therefore minimal movement of the bone fragments against each other provides an ideal environment for the soft tissue to recover.

Timing of CT scans should be carefully considered. In highly comminuted and displaced fractures of the tibial pilon performing a CT scan prior to final reduction or temporal external fixation will most likely offer little information regarding the fragment position and the surgical approaches needed. Tornetta et al. even concluded that after reviewing the CT scan the operative plan changed in 64% of the cases (38). Therefore, the motto of “span, scan, and plan” remains most advisable in order to get a full picture of the main fracture line, the fracture pattern and the number of fragments (26).

## Timing of Surgery

Commonly it is agreed upon that the state of the soft tissue dictates timing of surgery, but a clear guideline or set of criteria has not yet been established to help in the decision making.

In general most surgeons will agree that “wrinkling of the skin” seems to be the best indicator from operability, which in most cases will occur ~10–14 days after the trauma (33). In fractures with extensive soft tissue damage and subsequent formation of fracture blisters it has been suggested to postpone ORIF until reepithelization of the region is achieved. Furthermore, surgical approaches might be limited due to the location of the blisters (7).

Open fractures will need more careful consideration and might require multiple soft –tissue surgeries with VAC treatment. External fixation might also present a viable course of treatment, but in case ORIF is the desired treatment option the wound has to be sterile first (39).

## Approaches

There are several different ways to address the distal tibia. An extensive study of the fracture-lines and fragments via CT scans and/or 3D imaging should always dictate the approach used (40–42). The main focus should be on anatomical reduction of the articular surface. Furthermore, it might be necessary to adapt the used approach to soft tissue conditions, a.e. fracture blisters, open wounds, etc. (8). In complex fractures or fractures of both the tibia and the fibula combining approaches might be necessary. The minimal distance of the incisions used in order not to compromise blood flow to the soft tissue has been extensively discussed. In general a 7 cm skin bridge is deemed the save distance between two surgical incisions, but recent studies have shown that skin bridges of ~5–7 cm also seem to be associated with low complication rates (43). In small incisions as for example used in minimally invasive techniques the distance between the incisions can be even smaller (44). Furthermore, a recent retrospective study on 581 patients with surgically treated tibial pilon fractures shows that the approach chosen does not seem to have any effect on the rate of post-operative infections (45).

Various approaches to address tibial pilon fractures have been described in the recent past (46, 47). Which one to use and how to combine different approaches is up to the treating surgeon and should be planned on the precise analysis of the preoperatively conducted CT scans.

## Fixation of Fibula Fractures

Fibula fractures are present in up to 90% of all tibial pilon fractures (48). The topic of if and when to treat these fractures remains controversial (49–51).

In the acute trauma setting, ankle spanning external fixators should be applied to restore the tibial length and help to preserve the soft tissue. When the two –staged treatment protocol was first introduced, some authors suggested internal fixation of simple, non-comminuted fibula fractures simultaneously to mounting the temporary ankle spanning external fixator (28, 52, 53). They suggested that restoring the length of the fibula helps with the later realignment and reconstruction of the tibia especially in valgus deformation. Furthermore, fixation of the fibula might aid via indirect reduction of the Volkmann and Chaput fragment through ligamentotaxis, provided the anterior and posterior syndesmosis are intact (28, 35). Nevertheless, early fixation of the fibula requires a thorough understanding of the fracture pattern and an exact plan of treatment. The approach for fixation of the fibula needs to be chosen carefully in order not to interfere with later incisions needed for the final reduction of the tibia, a.e. the posterolateral approach (26). This can prove to be difficult as CT scans are often conducted after temporary external fixation so the further procedure might not be fully planned at the time of fibular fixation (54).

When it comes to definite treatment, recent studies suggest that not addressing the fibula fracture at all is beneficial in metaphyseal non-rotational distal tibia fractures. The benefits derived were lower rates of infection and fewer soft tissue problems and also a lower rate of non-union cases. Furthermore, fibula implants tend to cause irritation requiring implant removals and another surgery later on (53, 55). Modern therapeutical strategies, like intramedullary nailing or retrograde intramedullary screw fixation, may be beneficial in reducing the soft tissue problems, but problems of malrotation have to be considered and further discussed in the current literature (56, 57).

In a retrospective study Rouhani et al. found no difference in outcomes of tibial pilon fractures with or without fibula fixation at 2 years follow-up (51). Nevertheless, it is commonly agreed that fibula fixation is mandatory in metaphyseal fractures with syndesmotic injury (53).

## External Fixation as Definitive Treatment

The unacceptably high complication rate in the 1990's prompted the search for new definitive treatment methods.

From thereon, external fixators were not only used as a temporary fixation device. Especially in highly comminuted distal tibia fractures with maximal soft tissue damage, Gustilo type III –open fractures, highly contaminated wounds or significant comorbidities of the patient, ORIF is associated with a high risk of failure and complications. Therefore, external fixators can present a satisfying alternative form of definitive treatment (58).

External fixation methods can also be combined with limited ORIF or minimally invasive techniques, for example percutaneous K- wire or lag-screw constructs, to better reduce the articular surface (42, 59).

Several different techniques of external fixation are described in the literature. These include simple ankle spanning or ankle sparing bridging frames, circular frames or hybrid frames (37).

The most common complication associated with the use of external fixators in general is that of a pin tract infection, which can also lead to major deep infections namely septic arthritis and osteomyelitis (54).

In general, a basic distinction can be made between ankle sparing and ankle spanning fixator systems. Ankle sparing systems are beneficial for functional outcome as the movement in the ankle is not restricted. Additionally, full weight-bearing is possible especially in Hybrid-systems (60, 61).

Papadokostakis et al. compared ankle spanning and ankle sparing external fixator systems and found no significant differences in regards to the rate of infections, non-union and time to union, but ankle spanning systems had a significantly higher incidence of mal-union (61). Furthermore, in ankle spanning systems the functional outcome was also reported to be significantly lower in comparison to ankle sparing systems (62).

Multiple studies have furthermore compared the outcomes of ORIF and EF. The outcome seems to be similar in terms of early complications, but a significantly higher rate of superficial infection mostly due to pin tract infections were noted. However, the rate of deep infections did not vary significantly in ORIF and EF. The rate of mal union was significantly higher in EF than in ORIF, which is most likely due to the limited possibility of anatomical reduction in EF. Additionally, the functional outcome seems to be worse in EF than in ORIF, which was also attested to the reduced possibility of anatomical reconstruction of the articular surface (49, 63–66). Nevertheless, studies showed that fine wire EF and ORIF offer equivalent functional outcome in highly comminuted and severely displaced pilon fractures (58, 67–69).

The introduction of the Ilizarov external fixation method has presented further possibilities. Its use of tensioned transfixing wires offers the possibility of securely fixing small bone segments and therefore building a tight bone construct while still allowing axial micromovement, which is known to promote bone healing (70). Furthermore, its circular set up often entails that the ankle joint does not need to be transfixed and early movement is therefore possible enhancing blood flow to the injured cartilage (71). When significant bone loss is present, the Ilizarov fixation system can provide a tool for distraction osteogenesis (72).

**Figure 2:**
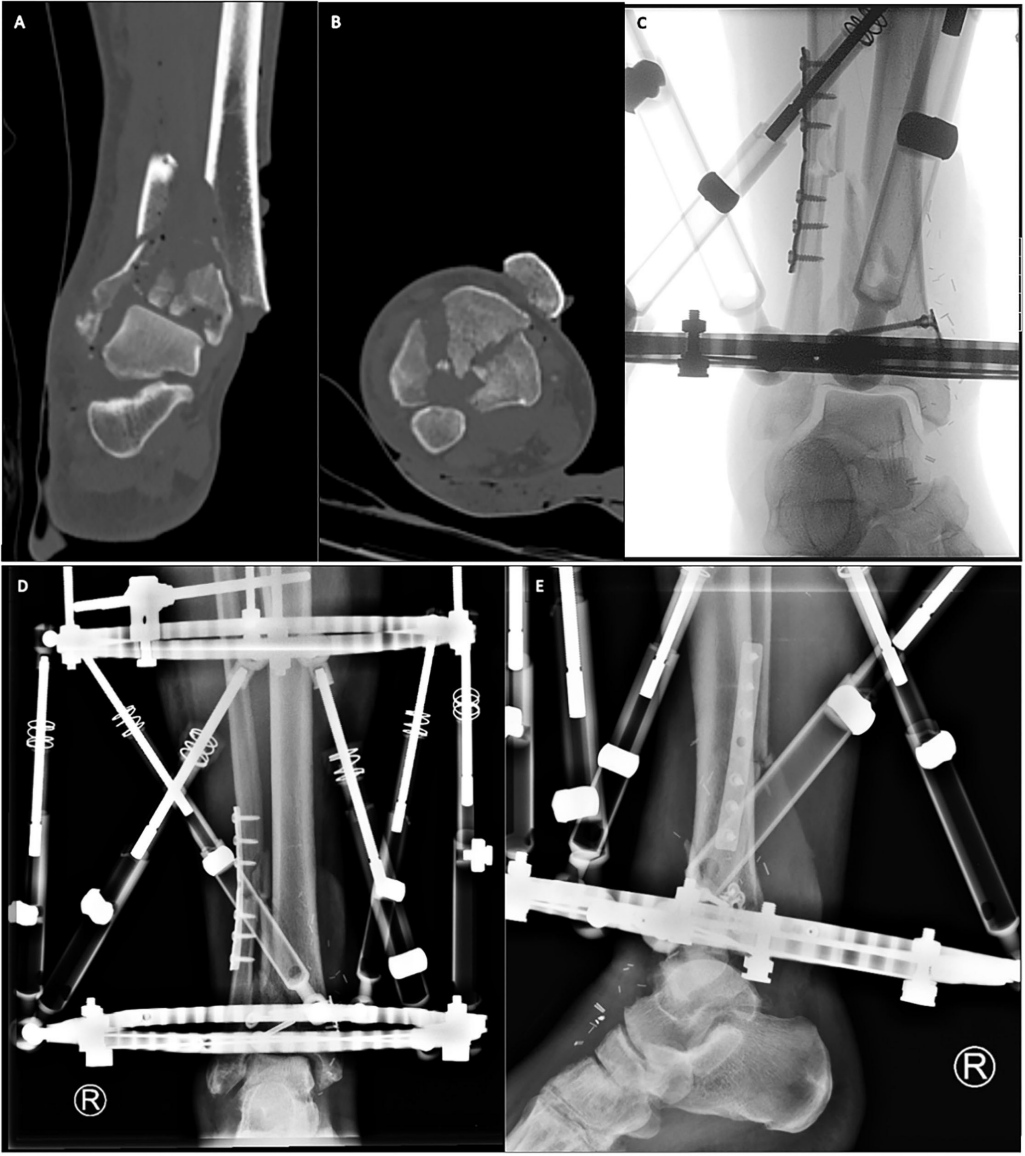
Primary Hybrid external fixation in combination with plate and lag screw osteosynthesis in a °II open tibial pilon fracture. We present a 55-year-old male, with a °II open tibial pilon fracture AO type 43-C3, who presented via the trauma room. After emergency preoperative CT scans [**(A)** coronal plane, **(B)** axial plane] temporary fixation and soft tissue conditioning was performed. The extensive soft tissue defect on the anteromedial side was covered with an arteria radialis microvascular free flap. Afterwards the comminuted fibula was restored in length and rotation and the articular surface of the tibial pilon was reconstructed using minimally invasive plating and lag screws [**(C)** Intraoperative fluoroscopy]. Finally, we stabilized the tibial pilon fracture using a hybrid circular frame with tensioned K-wires in the distal fragments and Schanz-screws in the proximal tibia [**(D)** Post-operative a.p. X-ray, **(E)** Post-operative lat. X-Ray].

Hybrid fixator systems consist of at least three tensioned K-wires, which are placed in the distal fragments of the fracture site and are connected via a circular frame (Figure 2). Mostly, hybrid external fixator systems can be used in ankle sparring technique and after some time full weight bearing is possible (60).

Nowadays external fixation methods for a primary definitive course of treatment is mainly indicated in tibial pilon fractures, where the risk for severe complications due to a poor soft tissue envelope, grade III open fractures or severe comorbidities of the patients is too great (72).

## Open Reduction and Internal Fixation (ORIF)

Nowadays a staged protocol is the main choice for ORIF in the treatment of tibial pilon fractures. The main goals are the anatomical reconstruction of the articular surface and the restoration of the correct rotational alignment to achieve best functional results (54).

Prior surgery, the CT scans should be analyzed meticulously to determine the approaches needed to address the main fragments. The three main columns, the anterolateral (Chaput), the posterolateral (Volkmann) and the medial column, should be assessed consecutively. Therefore, the chosen approaches should enable direct visualization of the main fracture zone and the joint block, while soft tissue should be preserved as well as possible (58).

**Figure 3:**
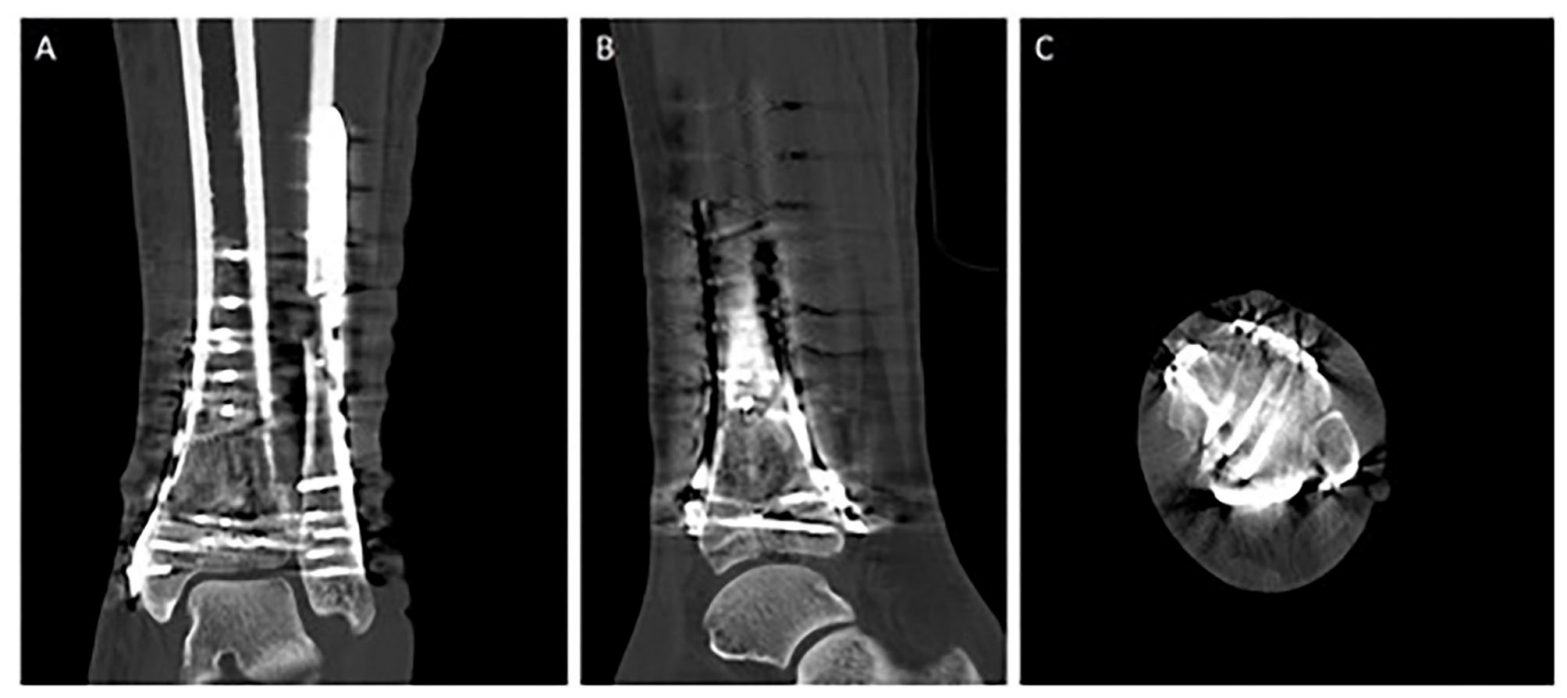
Triple plate ORIF of a tibial pilon fracture AO type 43-C3. Postoperative CT scans [**(A)** coronal plane, **(B)** sagittal plane, **(C)** axial plane] of a 49-year-old female patient following operative treatment of an AO type 43-C3 tibial pilon fracture, resulting from a hiking accident. As a first step, fracture reconstruction was started with a posterolateral approach to address the dorsal tibial component with a buttress plate. The comminuted fibula was restored in length and rotation afterwards. As a second step, the extensile approach was used to address the anterior and the anteromedial component with two angular stable plates.

The articular surface needs to be reduced sequentially, usually starting from lateral to medial and from posterior to anterior (Figure 3). The Volkmann fragment is often reduced first and used as a stabilizer so the rest of the joint block can be reduced around it (73). Reduction can temporarily be held using K- wires until the whole joint block is congruent. The final reduction is possible using lag screws, mini—fragment screws and anatomically formed locking or non–locking compression plates with a low profile in order to preserve the soft tissue as much as possible (36). Promising results using MIPO combined with lag screws have recently been published by Vicenti et al. (74).

In general fractures with valgus deformity often call for a anterolateral buttress plate, whereas varus deformities require a medial buttress (54). Posterior plating to address the posterior tibial column and prevent from tibial shortening needs to be achieved through posteromedial or posterolateral approaches (42).

In highly comminuted fractures with multiple fragments, it might be necessary to approach the fracture from anterior and posterior, sometimes even making two separate surgeries necessary. Furthermore, significant bone loss and comminution might call for bone grafting either from autologous or artificial bone material (26). Bone transport can also present a viable option when using circular frames (70). Furthermore, primary arthrodesis usually only serves as a salvage procedure in a very selected subgroup of patients (54, 75).

## Additional Treatment of Soft Tissue

In order to minimize the risk of soft tissue complications such as infection and skin necrosis, the introduction of the two–staged treatment protocol was the biggest advancement so far. Many further studies are currently being conducted on additional methods.

The need to thoroughly investigate the patient's history prior to surgery in order to identify possible risk factors such as diabetes, high blood pressure, immunodeficiency and nicotine abuse should always lay the common ground to preoperative care (7).

In recent years negative pressure wound therapy (NPWT) has become more popular. The NPWT system is applied to the wound after surgical wound closure instead of regular dressing of the wound. It provides a sealed space and decreases tension on the skin therefore increasing blood flow to the surgical site and reducing edema (76). Multiple studies showed promising results with a significant reduction of superficial and deep skin infections and a decreased incidence of wound dehiscence in high–risk injuries. No difference was found in terms of length of hospital stay (77, 78).

Further studies have suggested that the wound closure technique can be a significant modifiable factor in promoting wound healing. Better perfusion of the incision and least effect on cutaneous blood flow and therefore wound healing were seen in using the Allgöwer suture technique compared to vertical mattress (Donati) technique and stapling of the skin (79, 80).

While perioperative intravenous application of antibiotics belongs to the standard of care in surgical treatment of tibial pilon fractures, the benefit of local antibiotics is yet to be determined (33). The beneficial outcome of the perioperative use of vancomycin powder has been documented in spinal surgeries. O'Toole et al. are currently conducting a prospective randomized trial investigating whether the perioperative use of vancomycin powder could also significantly reduce the risk of infection after ORIF of tibial pilon fractures (81).

Historically, the infection rate following operative treatment of tibial pilon fractures used to be unacceptably high, with up to 50% (13, 24, 28). This changed significantly with the introduction of the two –staged treatment protocol of tibial pilon fractures, which led to reported infection rates of ~9–19% in recent studies (45, 82, 83). The most common complications after operative treatment of tibial pilon fractures are summarized in Table 1.

**Table 1:** Most common complications after operative treatment of tibial pilon fractures.

		**Carbonell-Escobar (82) 2017 (*n* = 92)**	**Harris 2006 (63) (*n* = 79)**	**Sirkin 1999 (28) (*n* = 56)**	**Van den Berg 2016 (84) (*n* = 118)**	**McCann 2011 (85) (*n* = 49)**
	Open fractures	22 (23.91%)	21 (27%)	22 (39%)	18 (15.3%)	3 (8.6%)
	Mean follow–up (in months)	39	26	N.A.	49.9	9.1
Soft tissue complications	Wound complications (i.e., necrosis, wound dehiscence, etc.)	7 (7.6%)	2 (2.5%)	6 (10%)	11 (9.3%) (Dehiscence 6, Necrosis 5)	1 (2%)
	Superficial infection	4 (4.3%)	2 (2.5%)	3 (5%)	12 (10.2%)	7 (14%)
	Deep Infection/Osteomyelitis	8 (8.7%)	1 (1.3%)	N.A.	4 (3.4%)	1 (2%)
Bone complications	Mal–union	N.A.	4 (5.1%)	2 (3%)	N.A.	1 (2%)
	Non–union	10 (10.9%) (3 infected non-unions)	2 (2.5%)	4 (6%)	5 (4.2%)	0 (0%)
Secondary operation	Revision surgery	5 (5.4%) (secondary arthrodesis)	8 (10%) (2 secondary arthrodesis)	0%	25 (21.2%)	5 (10%)
	Hardware removal	23 (25%)	N.A.	N.A.	52 (44.1%)	N.A.
Post-traumatic osteoarthritis		12 (13.0%)	31 (39%)	N.A.	N.A.	5 (10%)

Nevertheless, the overall poor functional outcome after high–energy tibial pilon fractures also proves the severity of the injury. In 2003 Pollak et al. conducted a large retrospective cohort analysis of pilon fractures using the SF- 36 score, which is based on a questionnaire on health—related quality of life and found that the scores were significantly lower in the injured group than in an age–matched uninjured population and even lower than in patients with chronic illnesses such as AIDS, diabetes or asthma (86). More recent studies confirmed these findings and furthermore conducted that 75% of patients, who were surgically treated for a tibial pilon fracture, reported noteworthy loss of function of the ankle joint and two third complained of suffering pain daily (4, 63, 83, 84, 87). Furthermore, return to work in these patients is generally low with only 57% having returned after 12 months (88). Another limiting factor is the high rate of post-traumatic arthritis after tibial pilon fractures. Harris et al. reported a rate of up to 50% at 2 years follow –up (63).

While age, gender and social status appear to have an effect on the functional outcome, the most important predictors for physical and functional outcome seem to be the extent of the fracture and soft tissue damage and further the quality of reduction, the congruence of the articular surface and the axial alignment of the tibiotalar joint (49, 54, 58, 63, 82, 88).

Although the change of treatment protocol, new surgical techniques and more advancement in terms of osteosynthesis hardware have led to more surgical options, the overall outcome after tibial pilon fractures is still only poor to moderate.

## Conclusion

Tibial pilon fractures are rare, but present an immense challenge for orthopedic surgeons. Preoperative planning includes CT scans and thorough investigation of the patient's history to identify possible risk factors, which is key to successful treatment. Special consideration and care should be taken in managing the fragile soft tissue envelope surrounding tibial pilon fractures. Choosing the right approach for each fracture pattern is important to get the best possible visualization and therefore be able to anatomically reduce the articular surface of the tibial pilon. With modern surgical techniques and hardware outcomes have improved, but are still only moderate with a high overall complication rate.

## Author Contributions

OM and MC: conception, design, data extraction, data analysis, data curation, writing, reviewing and editing, and project administration. PP and KH: data extraction and writing. KB, CK, and PB: writing and reviewing and editing. All authors contributed to the article and approved the submitted version.

## Conflict of Interest

The authors declare that the research was conducted in the absence of any commercial or financial relationships that could be construed as a potential conflict of interest.

## Publisher's Note

All claims expressed in this article are solely those of the authors and do not necessarily represent those of their affiliated organizations, or those of the publisher, the editors and the reviewers. Any product that may be evaluated in this article, or claim that may be made by its manufacturer, is not guaranteed or endorsed by the publisher.

## Abbreviations

ATA: Anterior tibial artery
ATFL: Anterior Talofibular Ligament
CFL: Calcaneofibular Ligament
CT: Computed-tomography
DPA: Dorsalis pedis artery
EF: External fixation
MIO: Minimally invasive osteosynthesis
MIPO: Minimally invasive plate osteosynthesis
NPWT: Negative pressure wound therapy
ORIF: Open reduction and internal fixation
PTA: Posterior tibial artery
PTFL: Posterior Talofibular Ligament.

## References

[1] Luo TD Eady JM Aneja A Miller AN. Classifications in brief: ruedi-allgower classification of tibial plafond fractures. Clin Orthop Relat Res. (2017) 475:1923–8. 10.1007/s11999-016-5219-z 28054323 PMC5449320

[2] Chen H Cui X Ma B Rui Y Li H. Staged procedure protocol based on the four-column concept in the treatment of AO/OTA type 43-C3.3 pilon fractures. J Int Med Res. (2019) 47:2045–55. 10.1177/0300060519836512 30890008 PMC6567754

[3] Mauffrey C Vasario G Battiston B Lewis C Beazley J Seligson D. Tibial pilon fractures: a review of incidence, diagnosis, treatment, and complications. Acta Orthop Belg. (2011) 77:432–40. 21954749

[4] Cutillas-Ybarra MB Lizaur-Utrilla A Lopez-Prats FA. Prognostic factors of health-related quality of life in patients after tibial plafond fracture. A pilot study injury. Injury. (2015) 46:2253–7. 10.1016/j.injury.2015.06.025 26115581

[5] Bartonícek J Mittlmeier T Rammelt S. [Anatomy, biomechanics and pathomechanics of the tibial pilon]. Ful Sprunggelenk. (2012) 10:3–11. 10.1016/j.fuspru.2012.01.017

[6] Luk PC Charlton TP Lee J Thordarson DB. Ipsilateral intact fibula as a predictor of tibial plafond fracture pattern and severity. Foot Ankle Int. (2013) 34:1421–6. 10.1177/1071100713491561 23720531

[7] Saad BN Yingling JM Liporace FA Yoon RS. Pilon fractures: challenges and solutions. Orthop Res Rev. (2019) 11:149–57. 10.2147/ORR.S170956 31576179 PMC6765393

[8] Krettek C Bachmann S. [Pilon fractures. Part 1: Diagnostics, treatment strategies and approaches]. Chirurg. (2015) 86:87–101. 10.1007/s00104-014-2895-7 25591416

[9] Renzi Brivio L Lavini F Cavina Pratesi F Corain M Bartolozzi P. The use of external fixation in fractures of the tibial pilon. Chir Organi Mov. (2000) 85:205–14. 11569083

[10] Topliss CJ Jackson M Atkins RM. Anatomy of pilon fractures of the distal tibia. J Bone Joint Surg Br. (2005) 87:692–7. 10.1302/0301-620X.87B5.15982 15855374

[11] Liporace FA Yoon RS. Decisions and staging leading to definitive open management of pilon fractures: where have we come from and where are we now? J Orthop Trauma. (2012) 26:488–98. 10.1097/BOT.0b013e31822fbdbe 22357091

[12] Ruedi T Matter P Allgower M. [Intra-articular fractures of the distal tibial end]. Helv Chir Acta. (1968) 35:556–82. 4974693

[13] Ruedi TP Allgower M. The operative treatment of intra-articular fractures of the lower end of the tibia. Clin Orthop Relat Res. (1979) 138:105–10. 376196

[14] Müller M Nazarian S Koch P Schatzker J. The Comprehensive Classification of Fractures of Long Bones, Vol. 1. Berlin: Springer-Verlag (1990).

[15] Meinberg EG Agel J Roberts CS Karam MD Kellam JF. Fracture and dislocation classification compendium-2018. J Orthop Trauma. (2018) 32(Suppl. 1):S1–170. 10.1097/BOT.0000000000001063 29256945

[16] Dirschl DR Adams GL. A critical assessment of factors influencing reliability in the classification of fractures, using fractures of the tibial plafond as a model. J Orthop Trauma. (1997) 11:471–6. 10.1097/00005131-199710000-00003 9334947

[17] Ramappa M Bajwa A Singh A Mackenney P Hui A Port A. Interobserver and intraobserver variations in tibial pilon fracture classification systems. Foot (Edinb). (2010) 20:61–3. 10.1016/j.foot.2010.06.002 20609577

[18] Swiontkowski MF Sands AK Agel J Diab M Schwappach JR Kreder HJ. Interobserver variation in the AO/OTA fracture classification system for pilon fractures: is there a problem? J Orthop Trauma. (1997) 11:467–70. 10.1097/00005131-199710000-00002 9334946

[19] Millar SC Arnold JB Thewlis D Fraysse F Solomon LB. A systematic literature review of tibial plateau fractures: what classifications are used and how reliable and useful are they? Injury. (2018) 49:473–90. 10.1016/j.injury.2018.01.025 29395219

[20] Dean DB. Field management of displaced ankle fractures: techniques for successful reduction. Wilderness Environ Med. (2009) 20:57–60. 10.1580/08-WEME-CON-240.1 19364168

[21] Ellanti P Hammad Y Kosutic D Grieve PP. Irreducible tibial pilon fracture caused by incarceration of the fibula in the tibial medullary canal. J Foot Ankle Surg. (2012) 51:362–4. 10.1053/j.jfas.2011.10.047 22153657

[22] Peterson ND Shah F Narayan B. An Unusual ankle injury: the bosworth-pilon fracture. J Foot Ankle Surg. (2015) 54:751–3. 10.1053/j.jfas.2014.09.016 25441267

[23] Cross WW Swiontkowski MF. Treatment principles in the management of open fractures. Indian J Orthop. (2008) 42:377–86. 10.4103/0019-5413.43373 19753224 PMC2740354

[24] Blauth M Bastian L Krettek C Knop C Evans S. Surgical options for the treatment of severe tibial pilon fractures: a study of three techniques. J Orthop Trauma. (2001) 15:153–60. 10.1097/00005131-200103000-00002 11265004

[25] Sitnik A Beletsky A Schelkun S. Intra-articular fractures of the distal tibia: current concepts of management. EFORT Open Rev. (2017) 2:352–61. 10.1302/2058-5241.2.150047 28932487 PMC5590002

[26] Stapleton JJ Zgonis T. Surgical treatment of tibial plafond fractures. Clin Podiatr Med Surg. (2014) 31:547–64. 10.1016/j.cpm.2014.06.002 25281515

[27] Newman SD Mauffrey CP Krikler S. Distal metadiaphyseal tibial fractures. Injury. (2011) 42:975–84. 10.1016/j.injury.2010.02.019 22073415

[28] Sirkin M Sanders R DiPasquale T Herscovici D Jr. A staged protocol for soft tissue management in the treatment of complex pilon fractures. J Orthop Trauma. (1999) 13:78–84. 10.1097/00005131-199902000-00002 10052780

[29] Borrelli J Jr. Ellis E. Pilon fractures: assessment and treatment. Orthop Clin North Am. (2002) 33:231–45. 10.1016/S0030-5898(03)00082-8 11832323

[30] McCormack RG Leith JM. Ankle fractures in diabetics. Complications of surgical management. J Bone Joint Surg Br. (1998) 80:689–92. 10.1302/0301-620X.80B4.0800689 9699839

[31] Teeny SM Wiss DA. Open reduction and internal fixation of tibial plafond fractures. Variables contributing to poor results and complications. Clin Orthop Relat Res. (1993) 292:108–17. 10.1097/00003086-199307000-00013 8519097

[32] McFerran MA Smith SW Boulas HJ Schwartz HS. Complications encountered in the treatment of pilon fractures. J Orthop Trauma. (1992) 6:195–200. 10.1097/00005131-199206000-00011 1602341

[33] Calori GM Tagliabue L Mazza E de Bellis U Pierannunzii L Marelli BM. Tibial pilon fractures: which method of treatment? Injury. (2010) 41:1183–90. 10.1016/j.injury.2010.08.041 20870227

[34] Anglen JO. Early outcome of hybrid external fixation for fracture of the distal tibia. J Orthop Trauma. (1999) 13:92–7. 10.1097/00005131-199902000-00004 10052782

[35] Patterson MJ Cole JD. Two-staged delayed open reduction and internal fixation of severe pilon fractures. J Orthop Trauma. (1999) 13:85–91. 10.1097/00005131-199902000-00003 10052781

[36] Tomas-Hernandez J. High-energy pilon fractures management: state of the art. EFORT Open Rev. (2016) 1:354–61. 10.1302/2058-5241.1.000016 28461913 PMC5367607

[37] Jacob N Amin A Giotakis N Narayan B Nayagam S Trompeter AJ. Management of high-energy tibial pilon fractures. Strategies Trauma Limb Reconstr. (2015) 10:137–47. 10.1007/s11751-015-0231-5 26407690 PMC4666229

[38] Tornetta P III Gorup J. Axial computed tomography of pilon fractures. Clin Orthop Relat Res. (1996) 323:273–76. 10.1097/00003086-199602000-00037 8625591

[39] Bear J Rollick N Helfet D. Evolution in management of tibial pilon fractures. Curr Rev Musculoskelet Med. (2018) 11:537–45. 10.1007/s12178-018-9519-7 30343399 PMC6220009

[40] Labronici PJ Junior AFM da Silva AAM da Silva PMG Serra M da Silva AF. CT mapping for complex tibial pilon fractures: understanding the injury pattern and its relation to the approach choice. Injury. (2021) 52(Suppl. 3):S70–6. 10.1016/j.injury.2021.04.064 34088468

[41] Zhao Y Wu J Wei S Xu F Kong C Zhi X. Surgical approach strategies for open reduction internal fixation of closed complex tibial Pilon fractures based on axial CT scans. J Orthop Surg Res. (2020) 15:283. 10.1186/s13018-020-01770-y 32718324 PMC7385877

[42] Hebert-Davies J Kleweno CP Nork SE. Contemporary strategies in pilon fixation. J Orthop Trauma. (2020) 34(Suppl. 1):S14–20. 10.1097/BOT.0000000000001698 31939775

[43] Howard JL Agel J Barei DP Benirschke SK Nork SE. A prospective study evaluating incision placement and wound healing for tibial plafond fractures. J Orthop Trauma. (2008) 22:299–305. 10.1097/BOT.0b013e318172c811 18448981

[44] Dresing K. [Minimally invasive osteosynthesis of pilon fractures]. Oper Orthop Traumatol. (2012) 24:368–82. 10.1007/s00064-012-0170-y 23015094

[45] Esposito JG van der Vliet QMJ Heng M Potter J Cronin PK Harris MB. Does surgical approach influence the risk of postoperative infection after surgical treatment of tibial pilon fractures? J Orthop Trauma. (2020) 34:126–30. 10.1097/BOT.0000000000001655 32084089

[46] Ren D Wang T Liu Y Liu P Wang P. Treatment of the tibial pilon fractures using the antero-medial fibula approach: ten case series. Medicine (Baltimore). (2020) 99:e20576. 10.1097/MD.0000000000020576 32664061 PMC7360227

[47] Wu D Peng C Ren G Yuan B Liu H. Novel anterior curved incision combined with MIPO for Pilon fracture treatment. BMC Musculoskelet Disord. (2020) 21:176. 10.1186/s12891-020-03207-3 32188447 PMC7081689

[48] Millington SA Grabner M Wozelka R Anderson DD Hurwitz SR Crandall JR. Quantification of ankle articular cartilage topography and thickness using a high resolution stereophotography system. Osteoarthritis Cartilage. (2007) 15:205–11. 10.1016/j.joca.2006.07.008 16949841

[49] Erichsen JL Andersen PI Viberg B Jensen C Damborg F Froberg L. A systematic review and meta-analysis of functional outcomes and complications following external fixation or open reduction internal fixation for distal intra-articular tibial fractures: an update. Eur J Orthop Surg Traumatol. (2019) 29:907–17. 10.1007/s00590-019-02368-9 30739163

[50] Faber RM Parry JA Haidukewych GH Koval KJ Langford JL. Complications after fibula intramedullary nail fixation of pilon versus ankle fractures. J Clin Orthop Trauma. (2021) 16:75–9. 10.1016/j.jcot.2020.12.025 33717942 PMC7920162

[51] Rouhani A Elmi A Akbari Aghdam H Panahi F Dokht Ghafari Y. The role of fibular fixation in the treatment of tibia diaphysis distal third fractures. Orthop Traumatol Surg Res. (2012) 98:868–72. 10.1016/j.otsr.2012.09.009 23153666

[52] Morrison KM Ebraheim NA Southworth SR Sabin JJ Jackson WT. Plating of the fibula. Its potential value as an adjunct to external fixation of the tibia. Clin Orthop Relat Res. (1991) 266:209–13. 10.1097/00003086-199105000-00032 2019053

[53] Torino D Mehta S. Fibular fixation in distal tibia fractures: reduction aid or nonunion generator? J Orthop Trauma. (2016) 30(Suppl. 4):S22–5. 10.1097/BOT.0000000000000695 27768629

[54] Zelle BA Dang KH Ornell SS. High-energy tibial pilon fractures: an instructional review. Int Orthop. (2019) 43:1939–50. 10.1007/s00264-019-04344-8 31093715

[55] Kurylo JC Datta N Iskander KN Tornetta P III. Does the fibula need to be fixed in complex pilon fractures? J Orthop Trauma. (2015) 29:424–7. 10.1097/BOT.0000000000000304 26295736

[56] Giordano V Boni G Godoy-Santos AL Pires RE Fukuyama JM Koch HA Giannoudis PV. Nailing the fibula: alternative or standard treatment for lateral malleolar fracture fixation? a broken paradigm. Eur J Trauma Emerg Surg. (2020) 47:1911–20. 10.1007/s00068-020-01337-w 32144445

[57] Kho DH Cho BK Choi SM. Midterm outcomes of unstable ankle fractures in young patients treated by closed reduction and fixation with an intramedullary fibular nail vs open reduction internal fixation using a lateral locking plate. Foot Ankle Int. (2021) 42:1469–81. 10.1177/10711007211017470 34184908

[58] Krettek C Bachmann S. [Pilon fractures. Part 2: Repositioning and stabilization technique and complication management]. Chirurg. (2015) 86:187–201. 10.1007/s00104-014-2917-5 25673229

[59] Mittlmeier T Wichelhaus A. [Treatment strategy and planning for pilon fractures]. Unfallchirurg. (2017) 120:640–7. 10.1007/s00113-017-0383-5 28717978

[60] Galante VN Vicenti G Corina G Mori C Abate A Picca G. Hybrid external fixation in the treatment of tibial pilon fractures: a retrospective analysis of 162 fractures. Injury. (2016) 47(Suppl. 4):S131–7. 10.1016/j.injury.2016.07.045 27484831

[61] Papadokostakis G Kontakis G Giannoudis P Hadjipavlou A. External fixation devices in the treatment of fractures of the tibial plafond: a systematic review of the literature. J Bone Joint Surg Br. (2008) 90:1–6. 10.1302/0301-620X.90B1.19858 18160490

[62] Endres T Grass R Biewener A Barthel S Zwipp H. [Advantages of minimally-invasive reposition, retention, and Ilizarov-(hybrid)fixation for pilon-tibial-fractures fractures with particular emphasis on C2/C3 fractures]. Unfallchirurg. (2004) 107:273–84. 10.1007/s00113-004-0742-x 15048331

[63] Harris AM Patterson BM Sontich JK Vallier HA. Results and outcomes after operative treatment of high-energy tibial plafond fractures. Foot Ankle Int. (2006) 27:256–65. 10.1177/107110070602700406 16624215

[64] Pugh KJ Wolinsky PR McAndrew MP Johnson KD. Tibial pilon fractures: a comparison of treatment methods. J Trauma. (1999) 47:937–41. 10.1097/00005373-199911000-00022 10568726

[65] Richards JE Magill M Tressler MA Shuler FD Kregor PJ Obremskey WT. External fixation versus ORIF for distal intra-articular tibia fractures. Orthopedics. (2012) 35:e862–867. 10.3928/01477447-20120525-25 22691658

[66] Wang C Li Y Huang L Wang M. Comparison of two-staged ORIF and limited internal fixation with external fixator for closed tibial plafond fractures. Arch Orthop Trauma Surg. (2010) 130:1289–97. 10.1007/s00402-010-1075-6 20182880

[67] Lim JA Thahir A Zhou AK Girish M Krkovic M. Definitive management of open pilon fractures with fine wire fixation. Injury. (2020) 51:2717–22. 10.1016/j.injury.2020.08.029 32859367

[68] Mehta N Graham S Lal N Wells L Giotakis N Nayagam S Narayan B. Fine wire versus locking plate fixation of type C pilon fractures. Eur J Orthop Surg Traumatol. (2021). [Epub ahead of print]. 10.1007/s00590-021-03048-3 34159481

[69] Daniels NF Lim JA Thahir A Krkovic M. Open pilon fracture postoperative outcomes with definitive surgical management options: a systematic review and meta-analysis. Arch Bone Joint Surg. (2021) 9:272–82. 10.22038/abjs.2020.53240.2641 34239954 PMC8221448

[70] Osman W Alaya Z Kaziz H Hassini L Braiki M Naouar N. Treatment of high-energy pilon fractures using the ILIZAROV treatment. Pan Afr Med J. (2017) 27:199. 10.11604/pamj.2017.27.199.11066 28904724 PMC5579433

[71] Leung F Kwok HY Pun TS Chow SP. Limited open reduction and Ilizarov external fixation in the treatment of distal tibial fractures. Injury. (2004) 35:278–83. 10.1016/S0020-1383(03)00172-4 15124796

[72] Quinnan SM. Definitive management of distal tibia and simple plafond fractures with circular external fixation. J Orthop Trauma. (2016) 30(Suppl. 4):S26–32. 10.1097/BOT.0000000000000694 27768630

[73] Assal M Ray A Stern R. The extensile approach for the operative treatment of high-energy pilon fractures: surgical technique and soft-tissue healing. J Orthop Trauma. (2007) 21:198–206. 10.1097/BOT.0b013e3180316780 17473757

[74] Vicenti G Bizzoca D Nappi VS Carrozzo M Delmedico M Solarino G. The impact of lag screw in the healing time of distal tibia fractures treated with minimally invasive plate osteosynthesis: a randomized clinical trial. Injury. (2020) 51(Suppl. 3):S80–5. 10.1016/j.injury.2020.02.042 32070556

[75] Zelle BA Gruen GS McMillen RL Dahl JD. Primary arthrodesis of the tibiotalar joint in severely comminuted high-energy pilon fractures. J Bone Joint Surg Am. (2014) 96:e91. 10.2106/JBJS.M.00544 24897748

[76] Stannard JP Volgas DA McGwin G III Stewart RL Obremskey W Moore T Anglen JO. Incisional negative pressure wound therapy after high-risk lower extremity fractures. J Orthop Trauma. (2012) 26:37–42. 10.1097/BOT.0b013e318216b1e5 21804414

[77] Wang C Zhang Y Qu H. Negative pressure wound therapy for closed incisions in orthopedic trauma surgery: a meta-analysis. J Orthop Surg Res. (2019) 14:427. 10.1186/s13018-019-1488-z 31829217 PMC6907184

[78] Brem MH Bail HJ Biber R. Value of incisional negative pressure wound therapy in orthopaedic surgery. Int Wound J. (2014) 11(Suppl. 1):3–5. 10.1111/iwj.12252 24851728 PMC7951022

[79] Shannon SF Houdek MT Wyles CC Yuan BJ Cross WW III Cass JR Sems SA. Allgower-donati versus vertical mattress suture technique impact on perfusion in ankle fracture surgery: a randomized clinical trial using intraoperative angiography. J Orthop Trauma. (2017) 31:97–102. 10.1097/BOT.0000000000000731 28129268

[80] Sagi HC Papp S Dipasquale T. The effect of suture pattern and tension on cutaneous blood flow as assessed by laser Doppler flowmetry in a pig model. J Orthop Trauma. (2008) 22:171–5. 10.1097/BOT.0b013e318169074c 18317050

[81] O'Toole RV Joshi M Carlini AR Murray CK Allen LE Scharfstein DO. Local antibiotic therapy to reduce infection after operative treatment of fractures at high risk of infection: a multicenter, randomized, controlled trial (VANCO study). J Orthop Trauma. (2017) 31(Suppl. 1):S18–24. 10.1097/BOT.0000000000000801 28323797

[82] Carbonell-Escobar R Rubio-Suarez JC Ibarzabal-Gil A Rodriguez-Merchan EC. Analysis of the variables affecting outcome in fractures of the tibial pilon treated by open reduction and internal fixation. J Clin Orthop Trauma. (2017) 8:332–8. 10.1016/j.jcot.2017.05.014 29062214 PMC5647682

[83] Boraiah S Kemp TJ Erwteman A Lucas PA Asprinio DE. Outcome following open reduction and internal fixation of open pilon fractures. J Bone Joint Surg Am. (2010) 92:346–52. 10.2106/JBJS.H.01678 20124061

[84] van den Berg J Monteban P Roobroeck M Smeets B Nijs S Hoekstra H. Functional outcome and general health status after treatment of AO type 43 distal tibial fractures. Injury. (2016) 47:1519–24. 10.1016/j.injury.2016.04.009 27129909

[85] McCann PA Jackson M Mitchell ST Atkins RM. Complications of definitive open reduction and internal fixation of pilon fractures of the distal tibia. Int Orthop. (2011) 35:413–8. 10.1007/s00264-010-1005-9 20352430 PMC3047643

[86] Pollak AN McCarthy ML Bess RS Agel J Swiontkowski MF. Outcomes after treatment of high-energy tibial plafond fractures. J Bone Joint Surg Am. (2003) 85:1893–900. 10.2106/00004623-200310000-00005 14563795

[87] Marsh JL Weigel DP Dirschl DR. Tibial plafond fractures. How do these ankles function over time? J Bone Joint Surg Am. (2003) 85:287–95. 10.2106/00004623-200302000-00016 12571307

[88] Bonato LJ Edwards ER Gosling CM Hau R Hofstee DJ Shuen A. Patient reported health related quality of life early outcomes at 12 months after surgically managed tibial plafond fracture. Injury. (2017) 48:946–53. 10.1016/j.injury.2016.11.012 28233519

